# Overexpression of *γ*-glutamylcysteine synthetase gene from *Caragana korshinskii* decreases stomatal density and enhances drought tolerance

**DOI:** 10.1186/s12870-021-03226-9

**Published:** 2021-10-01

**Authors:** Baiyan Lu, Xinjuan Luo, Chunmei Gong, Juan Bai

**Affiliations:** 1 College of Horticulture, Northwest A&F University, Yangling, 712100 Shaanxi China; 2 College of Life Sciences, Northwest A&F University, Yangling, 712100 Shaanxi China; 3 School of Life Sciences, East China Normal University, Shanghai, 200241 China

**Keywords:** Drought stress, Auxin signaling, Gamma-glutamylcysteine synthetase, Stomatal development

## Abstract

**Background:**

Gamma-glutamylcysteine synthetase (γ-ECS) is a rate-limiting enzyme in glutathione biosynthesis and plays a key role in plant stress responses. In this study, the endogenous expression of the *Caragana korshinskii* *γ-ECS* (*Ckγ-ECS*) gene was induced by PEG 6000-mediated drought stress in the leaves of *C. korshinskii*. and the *Ckγ-ECS* overexpressing transgenic *Arabidopsis thaliana* plants was constructed using the *C. korshinskii*. isolated *γ-ECS*.

**Results:**

Compared with the wildtype, the *Ckγ-ECS* overexpressing plants enhanced the γ-ECS activity, reduced the stomatal density and aperture sizes; they also had higher relative water content, lower water loss, and lower malondialdehyde content. At the same time, the mRNA expression of stomatal development-related gene *EPF1* was increased and *FAMA* and *STOMAGEN* were decreased. Besides, the expression of auxin-relative signaling genes *AXR3* and *ARF5* were upregulated.

**Conclusions:**

These changes suggest that transgenic *Arabidopsis* improved drought tolerance, and *Ckγ-ECS* may act as a negative regulator in stomatal development by regulating the mRNA expression of *EPF1* and *STOMAGEN* through auxin signaling.

**Supplementary Information:**

The online version contains supplementary material available at 10.1186/s12870-021-03226-9.

## Background

Water deficiency is one of the main reasons for poor plant performance and crop yields worldwide [1]. The global losses in crop yields due to drought totaled ~$30 billion in the past decade. Drought causes a wide range of changes including reduced leaf sizes, loss of root hairs, low water-use efficiency, and reduced photosynthesis [2–4]. Plants adopt multiple inherent strategies to (i) escape (acceleration of the reproductive phase before stress could impact its survival), (ii) avoid (increase internal water content), and (iii) tolerate drought (sustain growth with low internal water content during drought period) [5]. For example, redox homeostasis, root-associated microbiome, and transcription factors are all involved in plant resistance to drought. Oxidative damages can occur due to excessive reactive oxygen species (ROS) accumulated during water shortage [6]. ROS are the results of the partial reduction of atmospheric O_2_ and are metabolic products in all cells. Under physiological conditions, ROS function as intracellular messengers in redox signaling and many cellular processes, and there is a frail balance between ROS production and breakdown. Under stress, slight enhancement in ROS production is sensed by the plant as an alarm and triggers defense responses; while excessive accumulation of ROS and unrestricted oxidation will cause damages to DNA, proteins, and lipids, and ultimately cause cell death [7, 8].

The antioxidant glutathione (GSH) alleviates ROS damages, mainly through the redox signaling pathways [9–11]. The biosynthesis of GSH is tightly regulated by the rate-limiting enzyme gamma-glutamylcysteine synthetase (γ-ECS) [12]. In *Arabidopsis*, overexpression of *γ-ECS* (from *S. cerevisiae* strain S288C) led to a higher tolerance to heavy metals and the accumulation of arsenic and cadmium ions [13]. Over-expression of bacterial *γ-ECS* in poplar (*Populus tremula × P. alba*) and tobacco affected the photosynthesis and improved adaptability under mild drought and metal exposure [14–16]. In transgenic *Arabidopsis* overexpressing *γ-ECS* without seed vernalization, the level of flowering repressor *FLOWERING LOCUS* increased, suggesting that floral transition requires redox changes in GSH [17].

Stomata are vital to the drought tolerance of plants. Stomatal closure and the consequent limitation on CO_2_ fixation are ways of ROS accumulation under drought conditions [8]. As a gateway for water transpiration and photosynthesis CO_2_ exchange, stomata open and close pores according to the turgidity of a pair of guard cells [18, 19]. The development of stomata undergoes several stages. In *Arabidopsis thaliana,* stomatal lineage initiates from asymmetric divisions of meristemoid mother cells to generate meristemoids, which reiterate several asymmetric divisions to produce neighboring nonstomatal cells, before finally producing the guard mother cells (GMC). GMC undergoes a single symmetric division to produce a set of paired guard cells to form mature stomata [20]. These divisions require the basic helix-loop-helix (bHLH) transcription factors SPEECHLESS (SPCH), MUTE, and FAMA [21, 22]. The activities of SPCH, MUTE, and FAMA are regulated by an intrinsic signaling pathway including the secreted peptides, EPIDERMAL PATTERNING FACTORS (EPFs), receptor-like kinases ERECTA (ER) family, receptor-like protein TOO MANY MOUTHS (TMM) and MAPK cascades [20, 23, 24]. EPF1 and EPF2 expressed in the epidermis are negative regulators of stomatal development in *Arabidopsis.* Their activities depend on the TMM and ER family receptor kinases. They are genetically upstream of the genes for TMM, ER family, and the MAPKs cascades [25, 26]. EPF2 is primarily perceived by ER and the co-receptor TMM to inhibit stomatal development [27]. TMM is a negative regulator of stomatal formation in cotyledons and leaves [28, 29]. Transgenic *Arabidopsis*, tomato, and rice overexpressing ER showed improved heat tolerance and increased biomass in the greenhouse and field tests [30]. STOMAGEN, which is expressed in mesophyll and is a member of the EPFL family of proteins (EPF-LIKE9) [31], migrates to the epidermis where it is proposed to positively regulate stomatal density and stomatal index by competitively inhibiting TMM-mediated signaling [32]. Auxin signaling is a fundamental part of many plant growth processes and stress responses and operates through auxin/indole-3-acetic acid (Aux/IAA) protein degradation and the transmission of the signal via auxin response factors (ARFs) [33–35]. ARF5 directly binds to the *STOMAGEN* promoter to inhibit its expression, suggesting auxin negatively regulates stomatal development through ARF5 repression of the mobile peptide gene *STOMAGEN* in the mesophyll [36]. It has been shown that guard cells accumulate more GSH than other epidermal cells [37]. However, the precise function of GSH remains uncharacterized.

*C. korshinskii* is a leguminous shrub with strong tolerance to drought, cold, salt, and other biotic and abiotic stresses, and is widely distributed in arid and semiarid areas in the wilderness of Northwestern China [38]. Here, we isolated *γ-ECS* from *C. korshinskii* and characterized its role in mediating drought stress tolerance. The results suggest that *Ckγ-ECS* may participate in the negative regulation of auxin-mediate signaling to regulate stomatal development.

## Results

### The stomatal density of *C. korshinskii* leaves decreased with drought

**Fig. 1:**
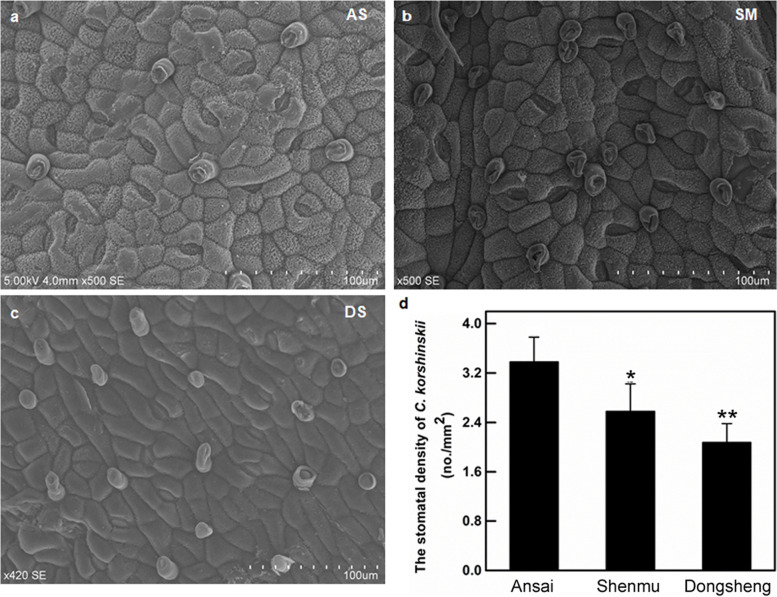
The stomatal density of *Caragana korshinskii* with different precipitation areas from Loess Plateau in China. Scanning electron micrographs of *C. korshinskii* leaves in Ansai, Shenmu, Dongsheng (**a**-**c**). The stomatal density of the *C. korshinskii* leaf with three sites (**d**). The data were shown above as means±SD (*n* ≥ 3). * and ** indicate that compared with Ansai, *P* < 0.05 and *P* < 0.01

The lowest stomata density was measured in leaves from Dongsheng, the driest site of the sampling sites. Under natural conditions, the stomatal density of *C. korshinskii* leaves decreased with intensified drought stress (*P* < 0.05, Fig. 1).

### Expression of stomatal development-related genes was changed after PEG 6000 treatment in *C. korshinskii*

**Fig. 2:**
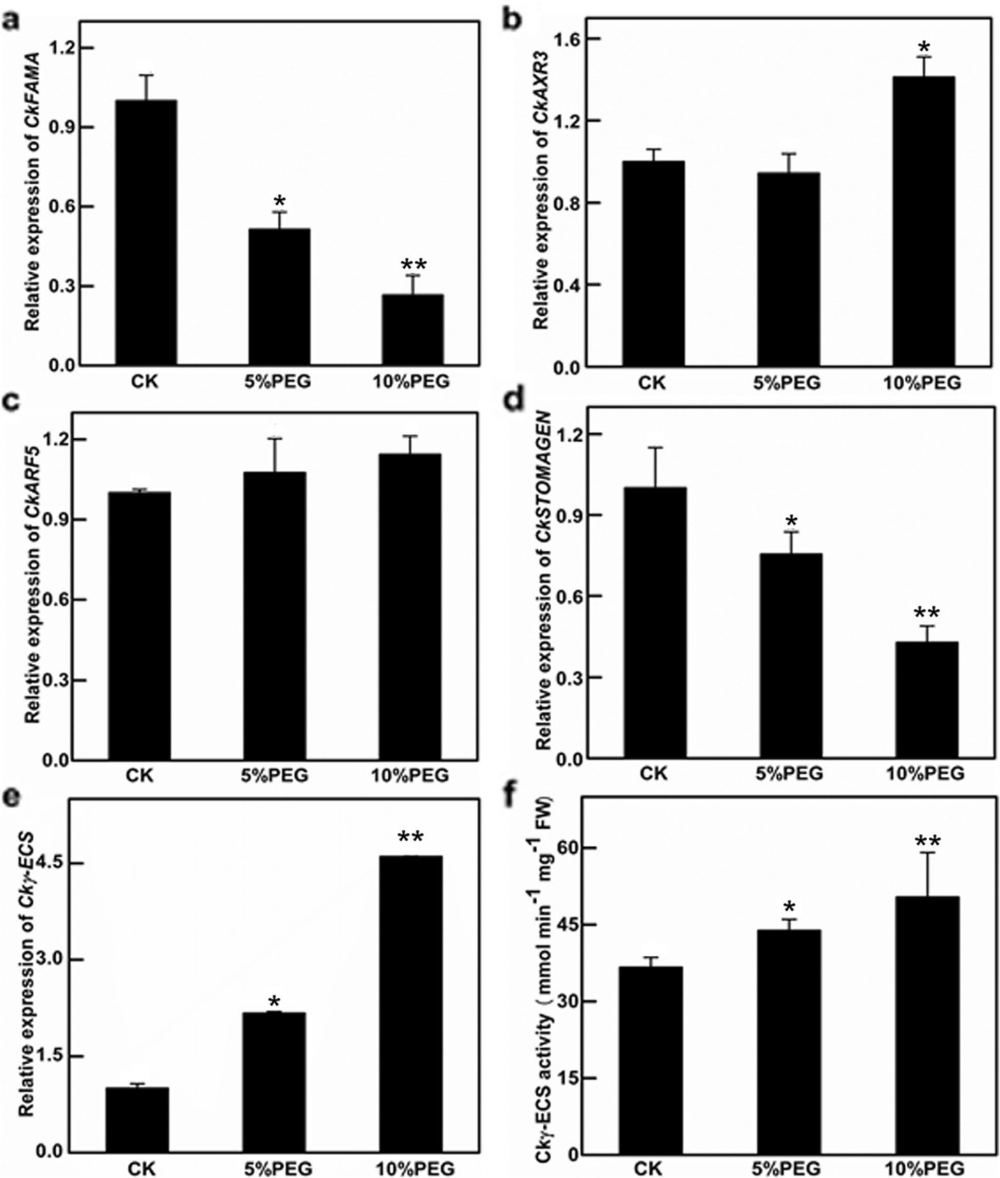
Relative genes expression and γ-ECS activity in *Caragana korshinskii* under different drought treatments. Relative genes expression of *C. korshinskii* (control, 5 and 10% PEG), including *CkFAMA*, *CkAXR3*, *CkARF5*, *CkSTOMAGEN,* and *Ckγ-ECS* (**a**-**e**).γ-ECS activity in *C. korshinskii* under different drought treatments (control, 5, 10 and 15% PEG 6000) (**f**). The data represented mean ± SD (*n* = 3), CK = control. * and ** indicate that compared with controls, *P* < 0.05 and *P* < 0.01

The relative expressions of stomatal development-related genes (*CkFAMA*, *CkSTOMAGEN, CkARF5,* and *CkAXR3)* were evaluated by qRT-PCR (Fig. 2). Under polyethylene glycol-6000 (PEG 6000) induced drought to *C. korshinskii* seeds, the expressions of *CkFAMA* and *CkSTOMAGEN* were decreased (Fig. 2a, d); the expression of *CkARF5* was increased slightly without a statistical difference (Fig. 2c), and the expression of *CkAXR3* was increased (Fig. 2b). The results suggested that auxin signaling may be involved in the stomatal development of *C. korshinskii*. Under drought. The expression of *Ckγ-ECS* and the γ-ECS activities also increased significantly with drought stresses (2.17-fold for 5% PEG and 4.6-fold for 10% PEG, Fig. 2e, f). These findings suggest that *Ckγ-ECS* is related to stomata development by auxin signaling to adapt to drought in *C. korshinskii.*

### Isolated *Ckγ-ECS* genes share a high identity with other Leguminosae plant γ-ECS

**Fig. 3:**
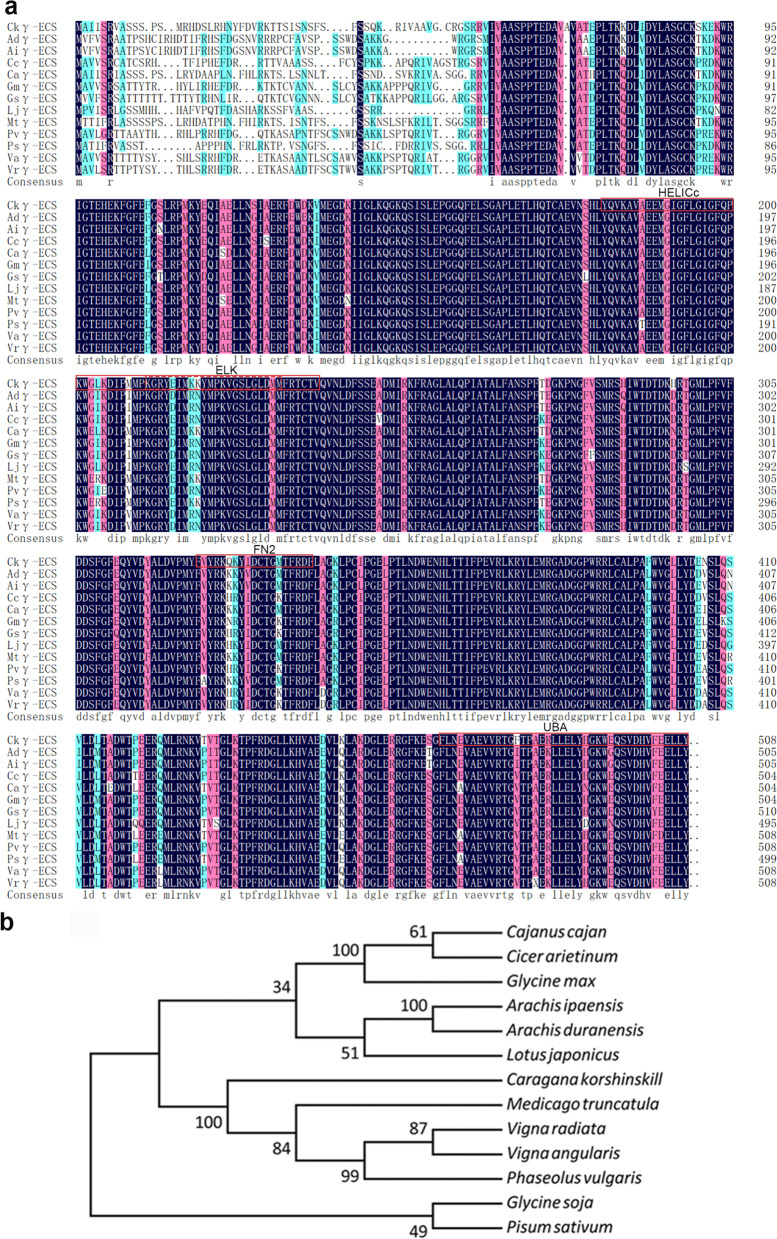
Sequence and phylogenetic analyses of Ckγ-ECS. Multiple alignments among the amino acid sequences of the putative protein of the Ckγ-ECS and its known homologs (**a**). Phylogenetic relationship among the putative Ckγ-ECS protein and other species (**b**)

The *Ckγ-ECS* gene was isolated from *C. korshinskii* using reverse-transcription PCR (RT-PCR), and the template was taken from the transcriptome data of *C. korshinskii* (Figs. S1 and S2). The *Ckγ-ECS* has an open reading frame of 1527 nucleotides, encoding 508 amino acids with an estimated molecular weight of 57.59 kDa and a calculated pI of 6.85. Sequence alignments indicated that the putative protein shared higher sequence identity with its homologous sequences, including (Fig. 3) *Cicer arietinum* (XP_004503722.1, 91%), *Medicago truncatula* (XP_003630512.1, 90%), *Cajanus cajan* (XP_020216402.1, 88%), *Pisum sativum* (AAF22137.1, 88%), *Lotus japonicus* (AAO45821.1, 87%), *Glycine max* (XP_003525397.1, 87%), *Phaseolus vulgaris* (XP_007160058.1, 86%), *Vigna angularis* (XP_017441981.1, 86%), *Glycine soja* (KHN16824.1, 86%), *Phaseolus vulgaris* (AAF22136.1, 86%), *Arachis duranensis* (XP_015955550.1, 85%), *Arachis ipaensis* (XP_016189524.1, 85%), *Vigna radiata* (NP_001304203.1, 85%) (Fig. 3a). The putative protein contained the HELICc, ELK, FN2, and UBA domains, which were highly conserved amongγ-ECS proteins (Fig. 3a). A phylogenetic analysis was performed to evaluate the evolutionary relationshipwith other species (Fig. 3b), and the results showed that Ckγ-ECS had a high degree of identity with the γ-ECS from other Leguminosae plants such as *Medicago truncatula*, *Phaseolus vulgaris*, *Vigna angularis*, *Vigna radiata* (Fig. 3b)*.*

### Generation and identification of Ckγ-ECS transgenic *Arabidopsis* lines

In order to verify the function of *Ckγ-ECS*, we generated transgenic *Arabidopsis* overexpressing the *Ckγ-ECS* cDNA without the termination under the control of the CaMV35S promoter and GFP fusion protein (Fig. S3a). Plants survived on the kanamycin selection medium and homozygous transgenic lines were obtained (Fig. S3b). After subsequent screening in T1 and T2 plant lines, homozygous T3 lines without resistance segregation on the selective medium were obtained and identified by the reverse transcription PCR amplification of the *Ckγ-ECS* gene using specific primers (Fig. S3c). The western blotting analysis showed that the Ckγ-ECS was expressed in the transgenic lines with an anti-GFP (Fig. S3d). T3 transgenic lines were used for further physiological studies.

### Stomatal density and aperture sizes decreased in *Ckγ-ECS* overexpressing *Arabidopsis*

**Fig. 4:**
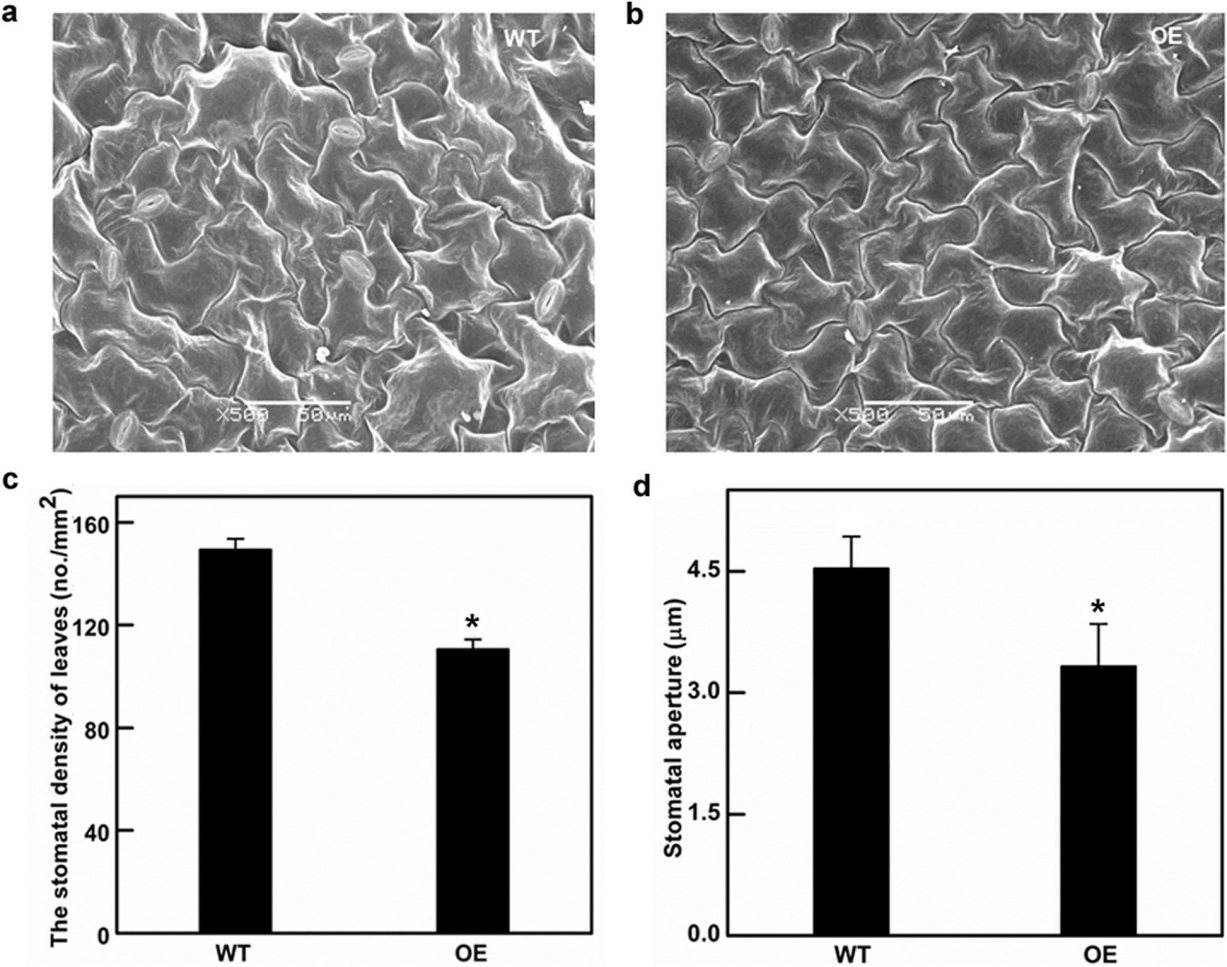
The effects of stomatal development on overexpressing plants. Scanning electron micrographs of *Arabidopsis* leaves (**a**, **b** 500X). Stomatal density and stomatal aperture of the *Arabidopsis* leaf between overexpression (OE) and wild type (WT) (**c**, **d**). * indicates *P* < 0.05

**Fig. 5:**
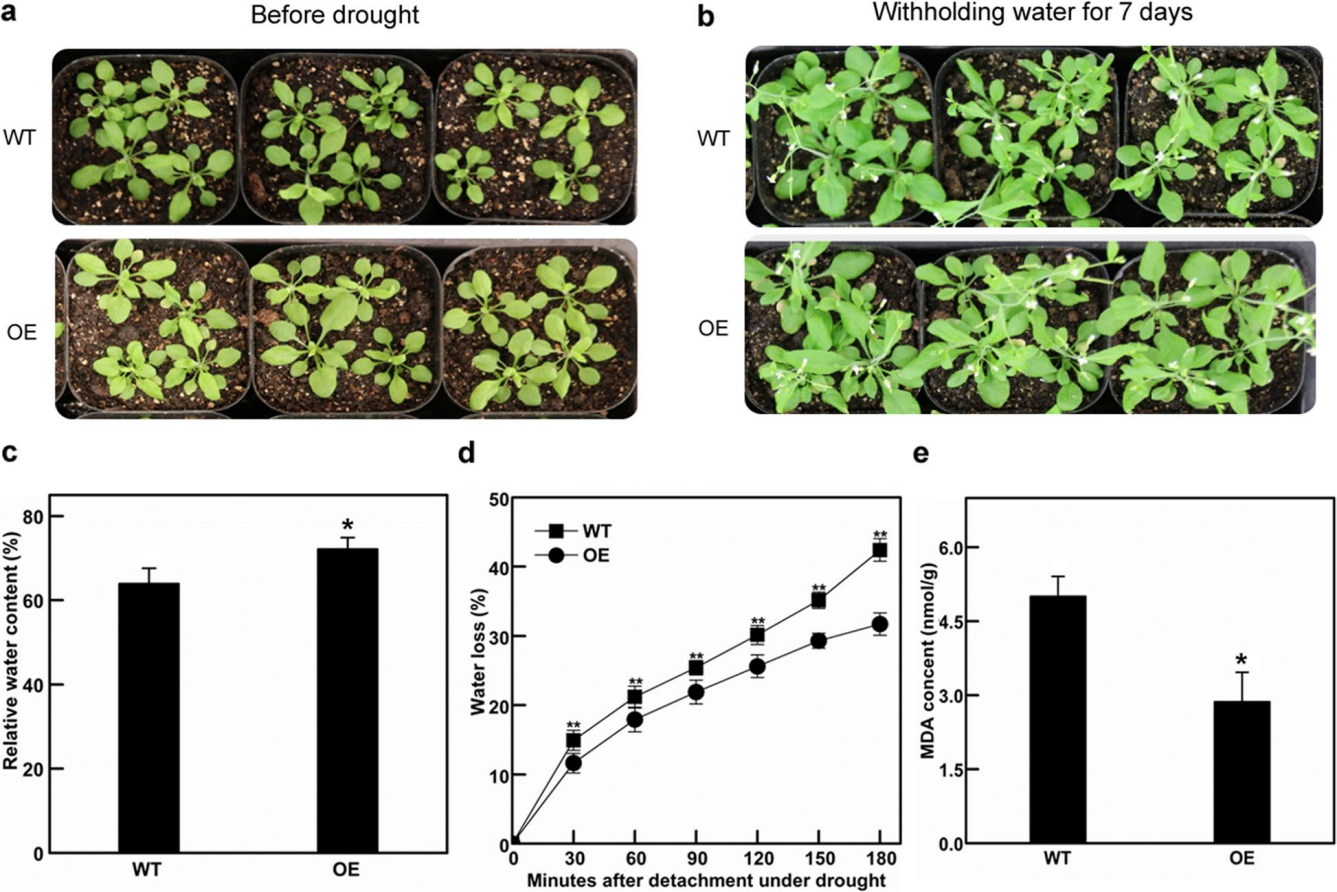
Drought tolerance of wild type (WT) and overexpressing transgenic (OE) plants. Phenotypic comparison of WT and overexpressing transgenic plants in response to drought stress before drought (**a**) and withholding water for 7 days (**b**). Relative water content in WT and overexpressing transgenic plants under drought stress (**c**). The rate of water loss in WT and overexpressing plants in response to dehydration (**d**). MDA content was measured in WT plants and transgenic lines after drought treatment (**e**). Data represent mean ± SD (*n* = 3).* and ** indicate that compared with WT, *P* < 0.05 and *P* < 0.01

**Fig. 6:**
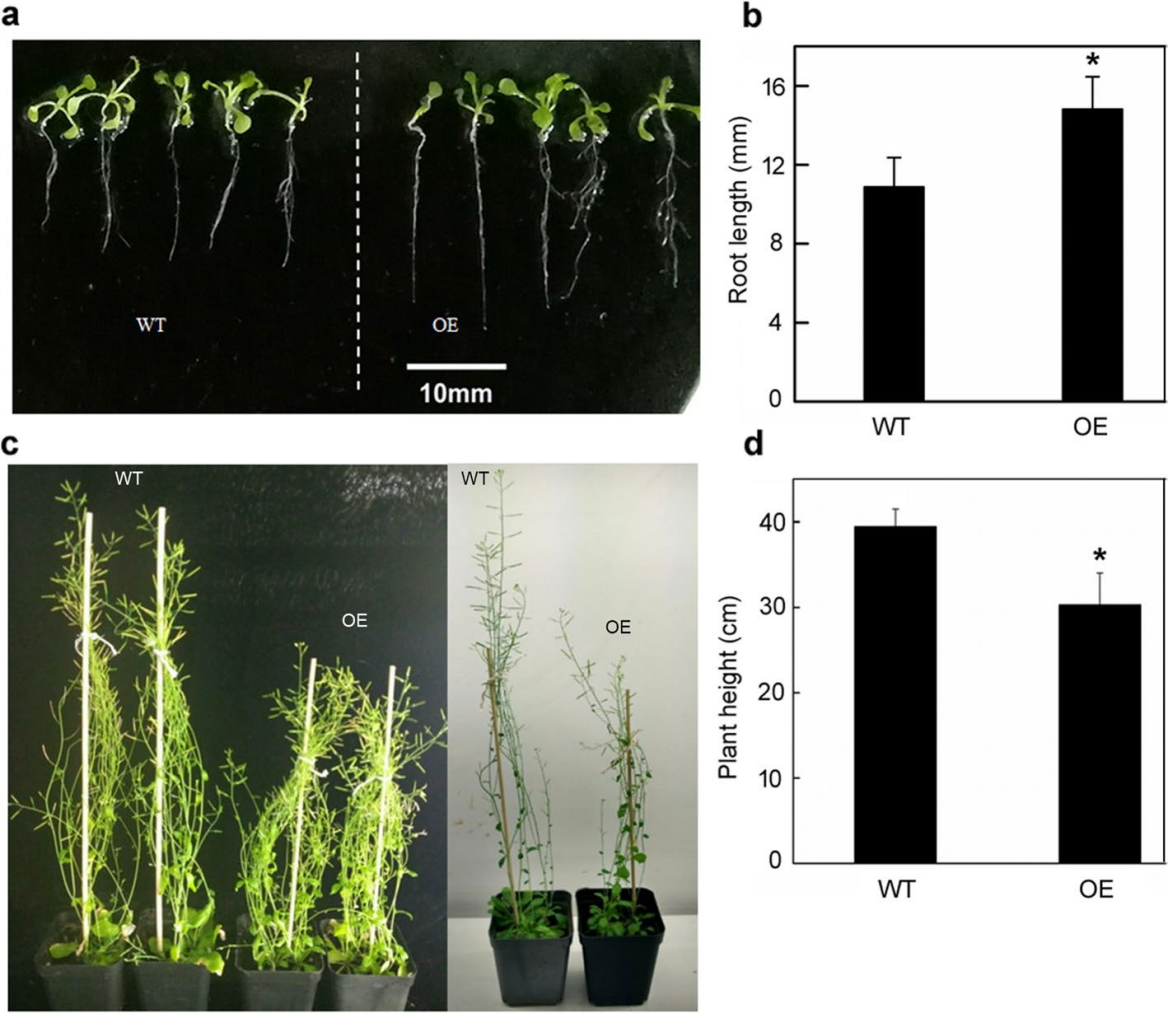
Main root length (mm, 7 days) (**a**, **b**) and plant height (cm, 60 days) (**c**, **d**) of *Arabidopsis thaliana* plants overexpressing Ckγ-ECS (OE) and wild type (WT) plants. * indicates *P* < 0.05

The *Ckγ-ECS* overexpressing *Arabidopsis* leaves were longer and narrower (Fig. S4), and their stomatal density and leaf aperture sizes were significantly reduced compared with those of WT plants (26.04 and 26.66% respectively, *P* < 0.05, Fig. 4). Overexpression of *Ckγ-ECS* led to a reduction in *Arabidopsis* stomatal density. The phenotypes of transgenic lines and WT plants under drought stress are shown in Fig. 5. The RWC of WT plants was significantly reduced (Fig. 5c). However, plants overexpressing *Ckγ-ECS* exhibited slower water loss in detached rosette leaves (Fig. 5d). Compared with WT, the MDA content of transgenic plants was significantly reduced (Fig. 5e). Compared with WT plants, the *Ckγ-ECS* transgenic *Arabidopsis* had a longer main root length withholding water for 7 days, and a shorter plant height at 60 days (Fig. 6). Together, the data indicate that overexpression of *Ckγ-ECS* in transgenic *Arabidopsis* can significantly improve drought tolerance.

### Relative expression of stomatal development-related genes was changed in *Ckγ-ECS* overexpressing *Arabidopsis*

**Fig. 7:**
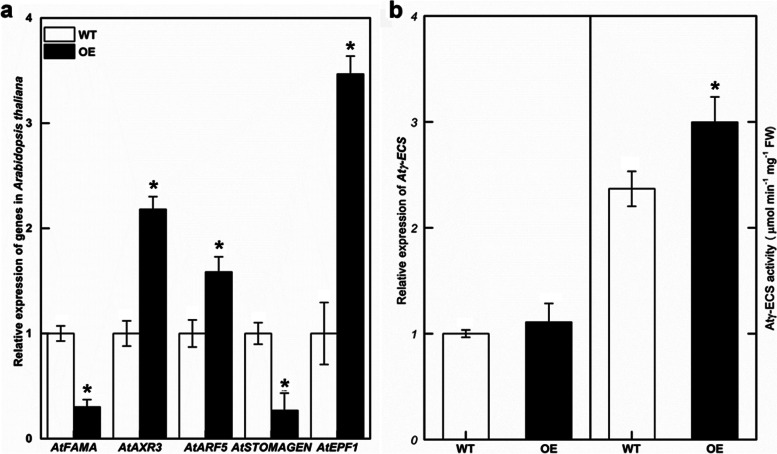
Relative genes expression and γ-ECS activity in *Arabidopsis thaliana*. Relative genes expression of *Arabidopsis thaliana* with overexpression lines and wild type (WT) (**a**). *γ-ECS* expression and γ-ECS activity in overexpression lines and WT (**b**). The data represented mean ± SD (*n* = 3), and * indicates *P* < 0.05

The levels of *AtFAMA* and *AtSTOMAGEN* mRNA in the transgenic plants were significantly lower than those in the WT (Fig. 7). The expressions of *AtAXR3*, *AtARF5* and *AtEPF1* in the transgenic plants increased significantly. There was no significant difference in the expression of *Atγ*-ECS between WT and transgenic plants (Fig. 7b), suggesting that overexpression *Ckγ-ECS* did not affect the expression of endogenous genes. However, γ-ECS activity in transgenic *Arabidopsis* was significantly increased (Fig. 7b).

## Discussion

We examined the effects of a GSH rate-limiting enzyme γ-ECS on the plant drought tolerance both in *C. korshinskii*. and in Ckγ-ECS transgenic *Arabidopsis* lines, and revealed that *Ckγ-ECS* improved plant drought tolerance and the possible mechanism was stomatal development inhibition through regulating the auxin signaling (changed *EPF1* and *STOMAGEN* expressions). The γ-ECS gene isolated from *C. korshinskii* and the putative proteins shared a high identity with those from other Leguminosae species.

Stomata play crucial roles in plant responses to various abiotic stresses. Under the natural environment of the three sampling sites in the Loess Plateau, the stomatal density of *C. korshinskii* decreased with increased drought stress. Under drought treatment, the expressions of stomatal development positive regulators *CkFAMA* and *CkSTOMAGEN* in *C. korshinskii* were inhibited, and the expresson of auxin-response gene *CkAXR3* was enhanced (*CkARF5* was not enhanced). Furthermore, the expression of Ckγ-EC*S* and activity of γ-ECS were significantly increased under drought treatment. Previous study show that drought stress positively affect auxin-response genes (such as Aux/IAA, ARFs, Gretchen Hagen3 (GH3), small auxin-up RNAs, and lateral organ boundaries (LBD)), to regulate plant growth and development [35, 39–41], which was consistent with our observation. The results indicated that *Ckγ-ECS* may be related to the stomatal development in *C. korshinskii*. by ARFs to cope with drought.

In the *Ckγ-ECS* overexpressing *Arabidopsis thaliana,* the stomatal density and leaf aperture size was significantly reduced. In *Arabidopsis thaliana,* STOMAGEN positively and EPF1 and EPF2 negatively regulate the leaf stomatal density, and they are perceived by a receptor complex composed of the receptor-like proteins TMM, ER, and ERL1/2 [42]. STOMAGEN positively regulates stomatal formation and SPCH protein levels, [42, 43] while EPF1 and EPF2 act antagonistically to STOMAGEN, they activate MAP kinase that phosphorylates and destabilizes the SPCH [42]. The effect of FAMA on epidermal stomatal density and frequency was consistent with that of STOMAGEN [44]. In this study, the expression of *AtSTOMAGEN* and *AtFAMA* significantly decreased while the expression of *AtEPF1* was enhanced in transgenic lines (Fig. 7a), which explained the reduction in stomatal density.

Many stress tolerance mechanisms are related to hormone signaling, such as BR [45], ABA [19], and auxin [36]. The phytohormone auxin signaling is a fundamental part of plant growth processes and stress responses [46]. Auxin accumulation activates signaling pathways and inducts auxin-responsive genes, which are mediated by the Aux/IAA and ARFs protein families [33–35]. Another report showed that auxin inhibited stomatal development through auxin response factors. ARF5 directly binds to the STOMAGEN promoter to inhibit its expression and thereby suppresses the development of stomat a[36]. As shown in Fig. 7a, ARF5 and STOMAGEN mRNA levels were opposite in overexpressing *Ckγ-ECS* plants. This result was consistent with a previous study in which the transgenic line with overexpression of the *OsIAA6* gene improved tolerance to drought stress via the regulation of auxin biosynthesis genes [40]. In the present study, the expression of *AtAXR3* (an auxin-inducible gene, same as IAA17 protein) is promoted, in a large part, which owes to the genetical position of AXR3 in upstream of the YDA MAP kinase cascade to regulate stomata formation in response to light and auxin signals [47]. There are some Aux/IAA members induced in different plants under drought. *SbIAA8*, *SbIAA11*, *SbIAA 22* in leaves and *SbIAA23* in roots were significantly up-regulated exposed to drought conditions in *Sorghum bicolor* [39]. 15 (OsIAA*1*, *2*, *4*, *6*, *7*, *9*, *13*, *16*, *18*, *19*, *20*, *21*, *22*, *27*, and *30*) genes were induced by drought treatment in rice [48]. Furthermore, γ-ECS activity in transgenic plants of overexpressing *Ckγ-ECS* was significantly increased (Fig. 7b), in line with the previous report [16]. Previous studies indicated that overexpression of the *γ-ECS* gene enhanced tolerance to drought in poplars [16]. These results suggested that γ*-* ECS could regulate stomatal development by mediating the auxin signaling pathway.

Under drought stress, *Ckγ-ECS* overexpressing plants grew better than wild-type plants (Fig. 5b), they had higher RWC, lower water loss, and lower MDA content (Fig. 5c-e). RWC and water loss are typical phenotypic and physiological parameters used to assess the water status of plants under drought stress [49, 50]. MDA content in tissues can be used as a biomarker to estimate the degree of lipid peroxidation and tolerance to oxidative damage caused by dehydration stress [51]. These results indicated that overexpression of *Ckγ-ECS* can enhance plant resistance and tolerance to drought stress.

## Conclusions

We cloned and characterized the *Ckγ-ECS* gene from *C. korshinskii*. The overexpression of *Ckγ-ECS* resulted in increased tolerance to drought stress in transgenic *Arabidopsis* lines. The function of *Ckγ-ECS* may be to negatively regulate stomatal development in response to drought by affecting the level of auxin-related genes. However, a detailed molecular mechanism by which *Ckγ-ECS* regulates stomatal density to adapt to drought remains unclear and requires further study.

## Methods

### Plant materials and growth conditions

The three sampling sites selected for natural *C. korshinskii* seeds were Ansai, Shenmu, and Dongsheng (with the annual precipitation and relative humidity of 506.5 mm, 60.8%; 440.8 mm, 54%; and 325.8 mm, 48.5%; respectively; Data available online (http://data.cma.cn/data/cdcdetail/dataCode/A.0029.0004.html) or in the provincial Statistical Yearbook published by the Statistics Bureau), along a precipitation gradient from south to north across the Loess Plateau in north-west China. The sampling procedures were carried out in accordance with the institute guidelines by professor Fang Xiangwen from Lanzhou University. Sampled *C. korshinskii* seeds were grown in sterile distilled water under 16 h of light at 25 °C and 8 h of dark at 22 °C. The drought treatments included mock, 5% (w/v) PEG 6000, and 10% PEG 6000. After 20 days of growth, plants were sampled and stored with different treatments at − 80 °C for future tests.

Seeds of *Arabidopsis thaliana* (Col-0) were produced in our lab in the Northwest A&F University and maintained by Dr. Yang Yazhou. The wild-type *Arabidopsis thaliana* (WT, ecotype Col-0) and overexpressing (OE) plants were germinated under long-day conditions (16 h/8 h, day/night cycles) at a controlled temperature (20 °C, day or night) and relative air humidity of 65%. These plants were grown in plastic square pots (6.8 cm × 6.8 cm × 7.8 cm) filled with soil (Pindstrup Sphagnum Moss Peat, Ryomgaard, Denmark) in a growth chamber. After 4 weeks, shoots were collected and stored in liquid N2. Some *Arabidopsis* plants were grown in Petri dishes on half-strength Murashige and Skoog medium (1/2 MS medium, pH 5.8) with 1% sucrose and 0.8% plant agar on 90 mm circular plates and stored for 3 days in cold and dark to synchronize germination. Then, the plates were placed in a plant growth chamber with 16 h day photoperiod and 20 °C. After 10 days the shoots were collected and stored in liquid N_2_. After growing the plants in the soil for 4 weeks under normal conditions, stress treatment was started. Then, drought was induced by withholding water to both the overexpression plants and wild-type plants (all 12 replicates of each group were analyzed).

### Stomatal density

Leaf samples (width: 5 -7 mm) were fixed in 4% glutaraldehyde (Sigma, USA) at 4 °C overnight, and then washed four times with 0.1 M phosphate buffer (PBS, pH 6.8) for 10 min. The samples were dehydrated with a series of ethanol mixtures (30%, 50%, 70%, 80%, and 90% ethanol) for 15 min, and then washed twice with 100% ethanol and isoamyl acetate for 30 min, respectively. The stomatal density was observed by scanning electron microscopy (SEM6360LV). Image analysis was performed using ImageJ (NIH, Bethesda, MD, USA). All experiments were carried out with three biological replicates and three technical experiments.

### RNA extraction and real-time qRT-PCR analysis

Total genomic DNA was extracted from plant leaves using the DNA-quick Plant System (Tiangen, Beijing, China) according to the manufacturer’s instructions. Total RNA was isolated from leaves using Quick RNA Isolation Kit (Huayueyang Biological Technologies, Beijing, China) according to the manufacturer’s instructions. The pairs of primer specific for -ECS were in PCR analysis. The PCR program used was: 95 °C for 5 min; then 37 cycles of 94 °C for 30 s, 58 °C for 15 s and 72 °C for 2 min; with a final step at 72 °C for 10 min. The PCR process was performed with 1 unit of Phanta™ EVO HS Super- Fidelity DNA Polymerase (Vazyme, Nanjing, China), dNTP Mix (10 mM each), a pair of primers (10 μM each), 5 × EVO Buffer (with 10 mM MgCl_2_), DNA template, and ddH_2_O in a final volume of 50 μl. The PCR products were analyzed on 1% agarose gels stained with ethidium bromide (EB) and visualized using the Visible Imaging System (Bio-rad, USA). For RT-PCR analysis, 2 μg of each total RNA sample was treated with the Fast Quant RT Kit (with gDNase) according to the manufacturer’s instructions (Tiangen, Beijing, China).

Quantitative Real-time PCR used ABI StepOnePlus. The primers for the *Actin7* gene of Arabidopsis and *ubiquitin* gene of *C. korshinskii* were used as an internal control. Each reaction contained 7.5 μl of 2 × ChamQ SYBR qPCR Master Mix (Vazyme, Nanjing, China), 1.5 μl of diluted cDNA template, 200 nM primers and 0.3 μl 50 × ROX reference Dye, then ddH_2_O was added up to 15 μl. The reaction procedures were as follows: denature at 95 °C for 30 s, followed by 40 cycles of 95 °C for 10 s, 60 °C for 30 s, melting curve by 95 °C for 10 s, 60 °C for 60 s, 95 °C for 15 s. The relative expression of the genes was calculated using the relative 2^−ΔΔCT^ method. The primers used in this study are listed in Table 1.

**Table 1:** Gene-specific primers used for cloning and qRT-PCR

Gene	Forward primer (from 5′ to 3′)	Reverse primer (from 5′ to 3′)
*Ckγ-ECS*(cloning)	ATGGCTATCATCTCCCGA	ATAAAGCAACCTCAAATA
*Ckγ-ECS*	TGCGAGCTCATGGCTATCATCTCCCGA	CGGCTGCAGATAAAGCAATTCCTCAAATA
*qCkUB*	GACTTTGACCGGGAAGACCA	CACCACGAAGACGGAGCACA
*qCkFAMA*	TGAACGTAGGAACCAGGCA	AACAAGAGGAAGAGACCAAGAAG
*qCkAXR3*	CTGGAAGATGTTTGTGGAATCA	AGGGACATTTACATCGTCATTACAC
*qCkARF5*	GTCAGTGTGCCTCGTCGTCTC	TGTCCTCCCCGTTAATCTTCA
*qCkSTOMAGEN*	AATCCTCACAACCTCAAAGAGAACT	TGGGGTCATTTCCCTCCACAGG
*qCkγ-ECS*	AAGTTGGTTCTCTCGGGCT	CGGTGCTTATCAGTATCGGTC
*qAtActin7*	CACTACCGCAGAACGGGAAA	GCGATGGCTGGAACAGAACC
*qAtFAMA*	CAGCAACATCAACTCTCTCCTTCC	TCTGGTCTGGTTCAACGCAA
*qAtAXR3*	CTCTTTTACCATGGGCAAACATGGA	AGGGAACATAGTCCCAGCTATTCA
*qAtARF5*	TAATGGGGAATGGAGGTTTG	GTGTAGAGTTCTCAGGTTTAGCAG
*qAtSTOMAGEN*	TAGGGTCGACAGCACCAACTTGTAC	TCATTTCCTTCGACTGGAACTTGCT
*qAtERF1*	TTCTTCTCCTTGCCTTTTTCC	AACCTAGACCCAGCCACCTG
*qAtγ-ECS*	CTGTTAAGAGGAGTAAGAGAGGGC	CCAGAGGCAAGATAGGCAATG

### Isolation and sequence analysis of *Ckγ-ECS*

The primers for *Ckγ-ECS* were designed based on the sequences of transcriptome data (Fig. S1). The PCR product was cloned into the pGEM-T Easy Vector System (Promega, USA) using PCR primers (Table 1). The products were inserted into the SacI/PstI-digested pCAMBIA2301 binary vector (modified). After that, the plasmids were introduced into Agrobacterium strain GV3101. The putative amino acid sequences were deduced using BioEdit and ExPASy (https://web.expasy.org/protparam/). The multiple protein alignment was performed using Clustal X software (version 2.0). DNAMAN (version 5.2.2) was used for sequence assembling and SMART (http://smart.embl-heidelberg.de/smart/set_mode.cgi?NORMAL=1) was used for conserved domain analysis. The phylogenetic tree was constructed with the neighbor-joining (NJ) method for molecular evolutionary analysis using TreeView in MEGA (version 5.10).

### Construction of *Ckγ-ECS* transgenic *Arabidopsis* plants

The *35S: Ckγ-ECS-GFP* vector was introduced into *Arabidopsis* for overexpression studies. *Arabidopsis* transformation used the floral dip method [52]. After harvest, the T1 seeds were dried on allochroic silica gel at room temperature and germinated on 1/2 MS medium containing 50 μg ml^− 1^ carbenicillin to select transgenic seedlings. Seeds obtained from the primary transgenic lines were germinated on an antibiotic medium, and PCR analysis was performed on resistant plants. The surviving positive T1 plantlets were transferred to soil to harvest T2 seeds. T2 and T3 seeds were germinated as previously described. T3 seeds were harvested and used as the materials for all experiments in *Arabidopsis.*

### Detection of transgenic lines

The transgenic lines were detected by PCR and Western blotting. A pair of *Ckγ-ECS* primers were used to amplify specific sequences. The total protein of the transgenic line was extracted and incubated with anti-GFP antibodies (1:5000; Beijing) and HRP-conjugated IgG secondary antibodies (1:5000; Transgen, Beijing) to detect the GFP fusion protein.

### γ-ECS activity analysis

The excised leaves (0.5 g fresh weight) were ground in a mortar to a fine powder with extraction buffer (50 mM Hepes, pH 7.5, 5 mM MgCl_2_, 100 mM NaCl and 10% glycerol) [53]. The enzymatic activities of γ-ECS were determined spectrophotometrically at 25 °C by measuring the rate of ADP formation using a coupling assay of pyruvate kinase and lactate dehydrogenase [54]. The extraction buffer contained 50 mM Tris, pH 8.0, 500 mM NaCl, 25 mM imidazole, 5 mM MgCl_2_, 10% (v/v) glycerol and 1% (v/v) Tween 20. A common reaction mixture (0.5 ml) was used for both proteins and contained 100 mM Hepes (pH 7.5), 150 mM NaCl, 10 mM MgCl_2_, 2 mM sodium phosphoenolpyruvate (PEP), 0.2 mM NADH, 5 units of type III rabbit muscle pyruvate kinase and 10 units of type II rabbit muscle lactate dehydrogenase [54]. The decrease rate in A_340_ nm was tracked using a spectrophotometer (V-1100D, Mapada).

### Measurement of relative water content, leaf water loss, and MDA content

The WT and overexpressing lines under drought for 7 days were sampled to detect relative water content (RWC), water loss, and MDA content. The RWC was determined as described previously [50]. The water loss determination was carried out according to [55]. MDA was measured by thiobarbituric acid reactive substances (TBARS) assay. Samples of 0.5 g leaves were ground in liquid N_2_ and then were added to1.5 ml 20% trichloroacetic acid (TCA). The homogenate was centrifuged at 3000 r/min for 10 min. Then the supernatant was then added to 1.5 ml of 0.5% thiobarbituric acid (TBA). The supernatant was heated to 100 °C for 10 min. The supernatant of 2 ml was sampled for measurement, and 0.5% TBA was used as a control. The absorbance of the supernatant was measured at 450, 532, and 600 nm. The difference was used to calculate the amount of MDA using an extinction coefficient of 6.22 mm^− 1^ cm^− 1^.

### Statistical analysis

Data analysis was performed using SPSS 17 (SPSS Inc., Chicago, IL, USA). Data are presented as the mean ± standard deviation. Student’s t test and one-way analysis of variance (ANOVA) (LSD model) were used to test differences where it is appropriate. *P* < 0.05 was considered a statistically significant difference for two-tailed tests. All figures were plotted in Origin 9.0 (Northampton, MA, USA).

## Supplementary Information

**Additional file 1: Fig. S1.***Ckγ-ECS* Sequence in transcriptome data. **Fig. S2.** Cloning the *Ckγ-ECS* gene by PCR. **Fig. S3.** Detection of *Arabidopsis* transgenic lines overexpressing Ckγ-ECS. Schematic representation of constructs used for agroinfiltration (a). Screening Overexpressing plant from 1/2 MS medium with antibiotic (b). RT-PCR of amplification of Ckγ-ECS gene in Overexpressing *Arabidopsis* (c). The western blotting analysis in the transgenic lines (d). **Fig. S4.** Phenotype (a) and statistical analysis (b, c, and d) of leaf growth of transgenic lines and wild type on 1/2 MS medium. The data represented mean ± SD (*n* ≥ 3), WT = wild type, OE = overexpressing plants. * indicates that compared with WT, *P* < 0.05.

## Data Availability

Sequence information was submitted to NCBI GenBank with the accession number MH287046.

## Abbreviations

γ-ECS: Gamma-glutamylcysteine synthetase
Ckγ-ECS: *Caragana korshinskii* γ-ECS
ROS: Reactive oxygen species
GSH: Glutathione
GMC: Guard mother cells
bHLH: Basic helix-loop-helix
SPCH: SPEECHLESS
EPFs: EPIDERMAL PATTERNING FACTORS
ER: ERECTA
TMM: TOO MANY MOUTHS
Aux/IAA: Auxin/indole-3-acetic acid
ARFs: Auxin response factors
PEG 6000: Polyethylene glycol-6000
RT-PCR: Reverse-transcription PCR
GH3: Gretchen Hagen3
LBD: Lateral organ boundaries
WT, ecotype Col0: Wild-type *Arabidopsis thaliana*
OE: Overexpressing
PBS, pH 6.8: Phosphate buffer
SEM6360LV: Scanning electron microscopy
EB: Ethidium bromide
NJ: Neighbor-joining
PEP: Phosphoenolpyruvate
RWC: Relative water content
FW: Weight
TW: Turgid weight
DW: Constant weight
TBARS: Thiobarbituric acid reactive substances
TCA: Trichloroacetic acid
TBA: Thiobarbituric acid
ANOVA: Analysis of variance
WT: Wild type
OE: Overexpression
