# Supplementary material for: Overexpression of γ-glutamylcysteine synthetase gene from Caragana korshinskii decreases stomatal density and enhances drought tolerance
Source: BMC Plant Biol. 2021 Oct 1;21:444. doi: 10.1186/s12870-021-03226-9 (PMC8485494; doi:10.1186/s12870-021-03226-9)

**Supporting information**


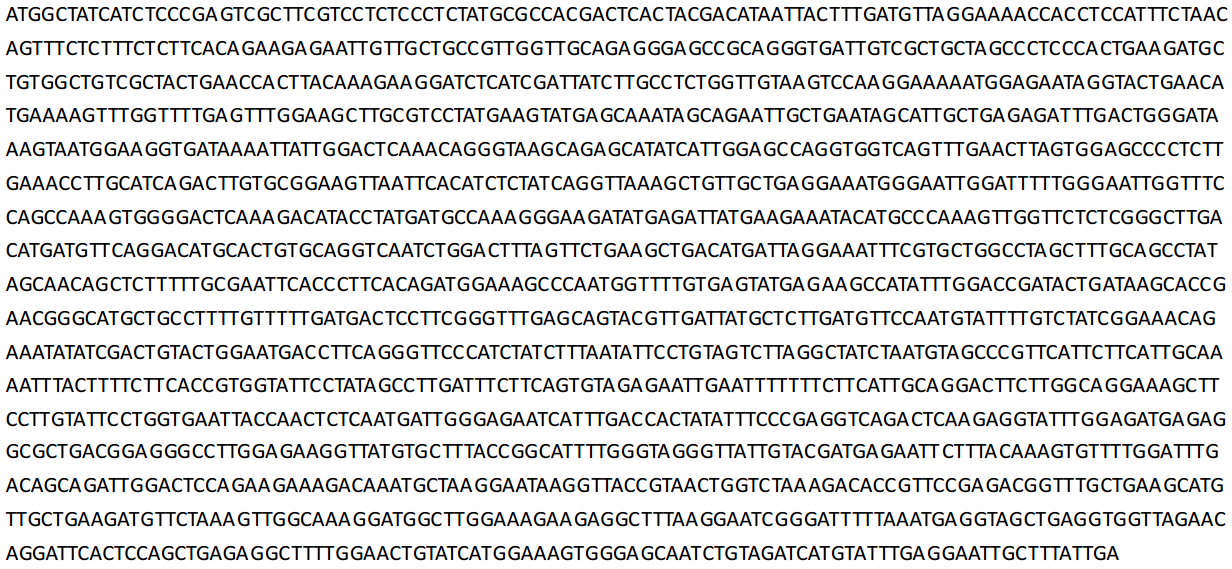


**Fig. S1** *Ckγ-ECS* Sequence in transcriptome data.


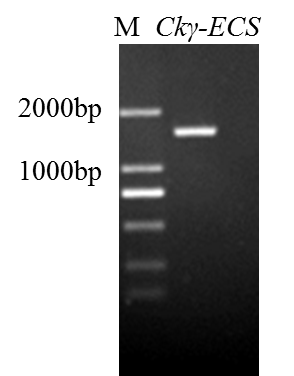


**Fig. S2** Cloning the *Ckγ-ECS* gene by PCR.

Fig S3 Detection of Arabidopsis transgenic lines overexpressing Ckγ-ECS. Schematic representation of constructs used for agroinfiltration (a). Screening Overexpressing plant from 1/2 MS medium with antibiotic (b). RT-PCR of amplification of Ckγ-ECS gene in Overexpressing Arabidopsis (c). The western blotting analysis in the transgenic lines (d).


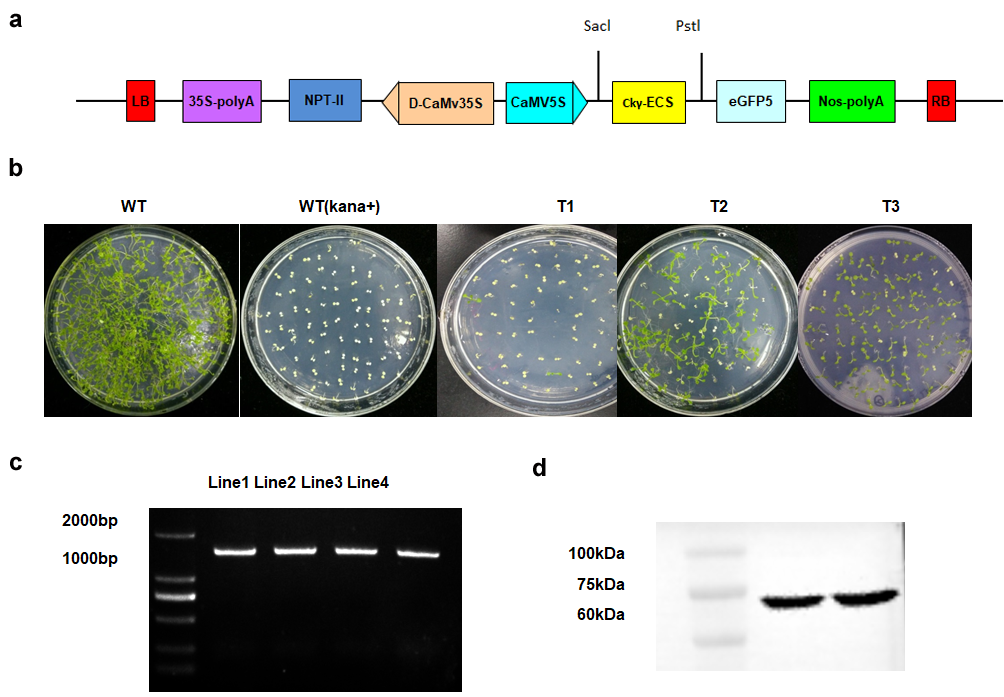


Fig. S4 Phenotype (a) and statistical analysis (b, c, and d) of leaf growth of transgenic lines and wild type on 1/2 MS medium. The data represented mean ± SD (n ≥ 3), WT = wild type, OE= overexpressing plants.  * indicates that compared with WT,  P<0.05.


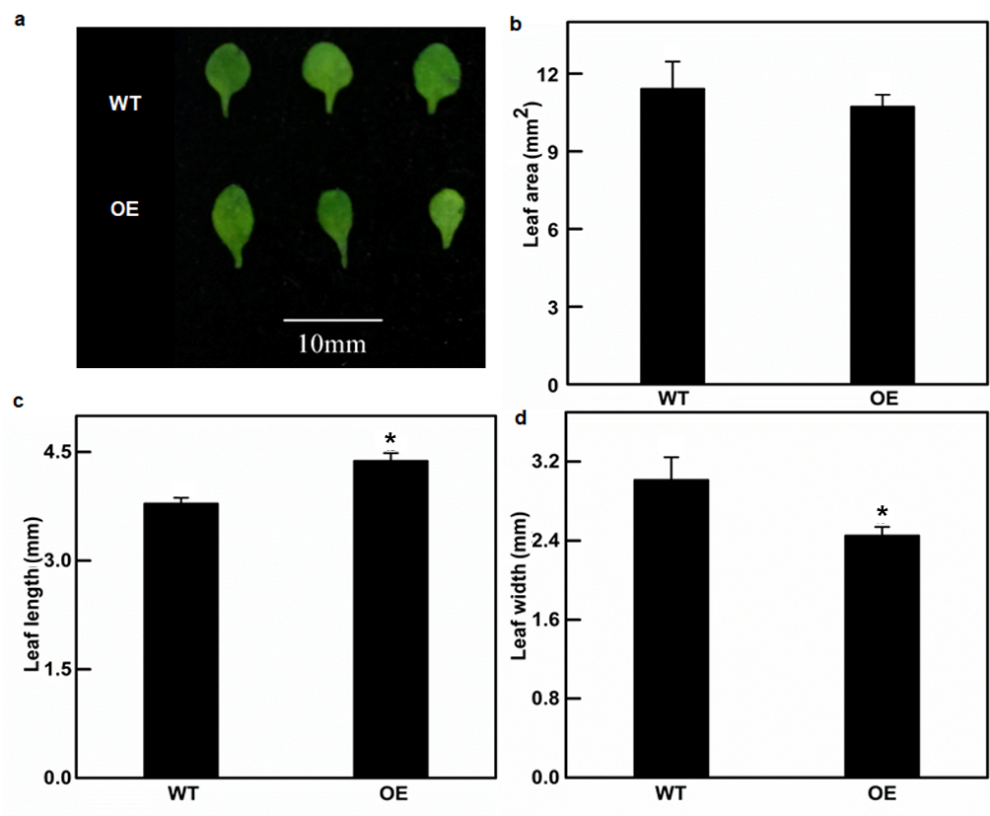

Supplement: Supplementary file 1 — Additional file 1: Fig. S1.Ckγ-ECS Sequence in transcriptome data. Fig. S2. Cloning the Ckγ-ECS gene by PCR. Fig. S3. Detection of Arabidopsis transgenic lines overexpressing Ckγ-ECS. Schematic representation of constructs used for agroinfiltration (a). Screening Overexpressing plant from 1/2 MS medium with antibiotic (b). RT-PCR of amplification of Ckγ-ECS gene in Overexpressing Arabidopsis (c). The western blotting analysis in the transgenic lines (d). Fig. S4. Phenotype (a) and statistical analysis (b, c, and d) of leaf growth of transgenic lines and wild type on 1/2 MS medium. The data represented mean ± SD (n ≥ 3), WT = wild type, OE = overexpressing plants. * indicates that compared with WT, P < 0.05. [file 12870_2021_3226_MOESM1_ESM.docx]
